# Incidence of and Risk Factors for Anti-PD-1/PD-L1- Associated Diarrhea and Colitis: A Retrospective Cohort Study of the Chinese Population

**DOI:** 10.3390/medicina61020353

**Published:** 2025-02-18

**Authors:** Wei Chen, Yan Wang, Mengyu Zhao, Hong Zhang, Ye Zong, Xinyan Zhao

**Affiliations:** 1 Beijing Digestive Disease Center, National Clinical Research Center for Digestive Disease, Beijing Key Laboratory for Precancerous Lesion of Digestive Disease, Department of Gastroenterology, Beijing Friendship Hospital, Capital Medical University, Beijing 100050, China; wchen2020yyyy@gmail.com (W.C.); zongye@ccmu.edu.cn (Y.Z.); 2 Liver Research Center, National Clinical Research Center for Digestive Disease, Key Laboratory on Translational Medicine on Cirrhosis, Beijing Friendship Hospital, Capital Medical University, Beijing 100050, China; wangyanpku@163.com (Y.W.); m18801123990@163.com (M.Z.); 3 Department of Pharmacy, Wenzhou Integrated Traditional Chinese and Western Medicine Hospital, Wenzhou 325000, China; 17858725926@163.com

**Keywords:** immune checkpoint-associated diarrhea and colitis (IMDC), incidence, risk factors, Chinese

## Abstract

*Background and Objectives*: The prevalence of and risk factors for immune checkpoint inhibitor-associated diarrhea and colitis (IMDC) in the Chinese population are unclear. This study aimed to estimate IMDC incidence and identify potential risk factors. *Materials and Methods*: We reviewed the electronic medical records from Beijing Friendship Hospital (2015–2022) to identify the patients treated with immune checkpoint inhibitors. The primary outcome was IMDC occurrence. The demographics, cancer type, baseline labs, and concurrent medications were analyzed. The univariable and multivariable analyses validated the associated factors. *Results*: Among 1186 patients (median follow-up: 217 days), the IMDC incidence was 4.6%, with colitis at 0.67%. Digestive system tumors increased the IMDC risk (OR 2.79, 95% CI 1.42–5.75, *p* = 0.004), while platinum agents decreased it (OR 0.41, 95% CI 0.21–0.78, *p* = 0.008). PPIs, antibiotics, NSAIDs, and glucocorticoids showed no significant association. Colitis was the third most common irAE, leading to ICI discontinuation (15.6%). *Conclusions*: IMDC prevalence is 4.6% in the Chinese population, the third most frequent irAE causing ICI discontinuation. Digestive tumors and platinum agents are risk and protective factors, respectively, while other medications show no significant impact.

## 1. Introduction

Immune Checkpoint Inhibitors (ICIs) that specifically target Cytotoxic T-Lymphocyte Antigen 4 (CTLA4), Programmed Death protein 1 (PD-1), and its Ligand (PD-L1) have significantly changed the treatment landscape for oncology and have shown promising outcomes for improving tumor prognosis. However, the activation of the immune system through these inhibitors has also introduced a new range of immune-related Adverse Events (irAEs) [1]. Among these irAEs, immune checkpoint inhibitor-associated diarrhea and colitis (IMDC) is particularly prevalent and frequently leads to treatment interruption, discontinuation, and even mortality [1,2,3].

Through a meta-analysis, the incidence of IMDC was estimated to be 7.97% to 10.69% for anti-PD-1/PD-L1 and 16.91% to 43.31% for anti-CTLA4 [4]. However, it should be noted that the primary epidemiologic studies included in this analysis primarily focused on the Caucasian population, leaving the epidemiological data for the Chinese population uncertain. A nationwide study found that patients who developed IMDC were more likely to be Caucasian (OR = 2.3) after adjusting by a multivariate regression [5], suggesting that the incidence of IMDC may vary among different races.

As for the risk factors for IMDC, according to Khoja, L et al., IMDC was found to be more prevalent in melanoma compared with non-small-cell lung cancer (NSCLC) [6], which indicates that the cancer type might influence the incidence of IMDC. In addition, previous studies have indicated that antibiotics [7,8], proton pump inhibitors (PPIs) [9], nonsteroidal anti-inflammatory drugs (NSAIDs) [10], and other anti-tumor medications [4] may serve as potential risk factors for IMDC. Nevertheless, this evidence was mainly based on retrospective studies, and their conclusions need to be verified by further studies, especially of the Chinese population.

Furthermore, within our clinical practice, it was observed that glucocorticoids were frequently employed for allergy prevention in the context of anti-tumor drug administration. As we know, the evident therapeutic efficacy of glucocorticoids for IMDC is confirmed, and they are preferred as the initial treatment option for high-grade IMDC [11,12]. However, the potential preventive impact of glucocorticoids on IMDC remains uncertain.

Consequently, this retrospective single-center cohort study was conducted utilizing the electronic medical records (EMRs) from Beijing Friendship Hospital, with the aim of providing a more precise estimation of IMDC incidence in the Chinese population and identifying the potential underlying risk factors.

## 2. Materials and Methods

### 2.1. Ethics Approval and Consent to Participate

This study’s protocol was approved by the Institutional Review Board of Beijing Friendship Hospital, Capital Medical University, and accompanied by a waiver for informed consent (No. 2020-P2-300-01).

### 2.2. Study Design and Patient Cohort

The electronic medical record (EMR) management system of Beijing Friendship Hospital was utilized to search for data from May 2015 to July 2022. The follow-up period commenced upon initiation of ICI treatments and concluded upon a diagnosis of IMDC or loss of follow-up.

The inclusion criteria encompassed adult patients with a solid tumor diagnosis who underwent ICI therapy at Beijing Friendship Hospital and had no prior history of chronic diarrhea. The exclusion criterion pertained to patients with insufficient follow-up duration.

### 2.3. Exposure Variables and Concomitant Medications

Basic demographic data, including age, gender, tumor type, history of smoking, and history of alcohol use were collected. Additionally, a history of autoimmune diseases, such as systemic lupus erythematosus, rheumatoid arthritis, ankylosing spondylitis, dermatomyositis, systemic sclerosis, sicca syndrome, vasculitis, IgG4-related disease, ulcerative colitis, Crohn’s disease, primary biliary cholangitis, primary sclerosing cholangitis, psoriasis, and autoimmune thyroiditis was also gathered. The cancer types of each patient were likewise collected.

The laboratory results closest to the commencement of immune checkpoint inhibitor (ICI) treatment were obtained, including white blood cell count (WBC), hemoglobin (HGB), platelet (PLT), serum creatinine (SCr), total bilirubin (TBil), albumin (Alb), C-reactive protein (CRP), and lactate dehydrogenase (LDH).

The utilization of PPIs, antibiotics, NSAIDs, and glucocorticoids from the initiation of ICI treatment until the end of the study was documented. Moreover, as per Bossi P. et al., various cytotoxic drugs and targeted drugs, such as 5-Fu, capecitabine, irinotecan, taxanes, anthracyclines, platinum salts, and tyrosine kinase inhibitors, were associated with diarrhea [13]. The medical history and concurrent use of cytotoxic or targeted drugs were meticulously examined for all patients who reported experiencing diarrhea. The identified drugs were mainly categorized as 5-Fu/capecitabine, irinotecan, taxanes, platinum, targeted drugs, and others.

Age and laboratory examination outcomes were recorded as quantitative data, whereas smoking history, alcohol consumption, autoimmune disease history, and concurrent medication usage were recorded as enumeration data.

### 2.4. Outcomes

The primary objective of this study was to evaluate the development of IMDC in patients receiving immune checkpoint inhibitors. Patients who experienced diarrhea following ICI treatment were meticulously assessed. The diagnosis of IMDC was established based on the following criteria: (1) the occurrence of diarrhea subsequent to ICI therapy; (2) the exclusion of other potential causes of diarrhea, including infection, tumor, other anti-tumor medications, enteral nutrition, and inflammatory bowel disease; and (3) the confirmation of colitis via endoscopic examination and pathological analysis. Secondary objectives included characterizing the clinical features of IMDC and determining the proportion of IMDC among all immune-related Adverse Events (irAEs) leading to discontinuation of ICI treatment.

### 2.5. Statistical Analysis

The study utilized the median (first and third quartiles) to describe continuous variables and numbers (percentages) for categorical variables. Normality tests were performed on continuous variables, revealing non-normal distributions (all *p*-values < 0.05). As a result, non-parametric tests were applied, with the Kruskal–Wallis test used for comparisons among three groups. Categorical variables were compared using the Chi-square test or Fisher’s exact test. In the univariable analysis, *p*-values were adjusted using the False Discovery Rate (FDR) correction. However, variables were included in the multivariable logistic regression analysis based on unadjusted *p*-values < 0.05, as the main purpose of the univariable analysis was to identify potential candidates for the multivariable model. FDR-adjusted data were also provided. The Log-rank test was conducted to compare the incidence rate of IMDC between patients with digestive system tumors and those with non-digestive system tumors. Data analyses were performed using R (version 4.2.1, https://www.R-project.org/ (accessed on 6 June 2023)). A two-sided *p*-value < 0.05 was considered statistically significant.

## 3. Results

### 3.1. Incidence of Immune Checkpoint Inhibitor—Associated Diarrhea and Colitis (IMDC)

**Figure 1:**
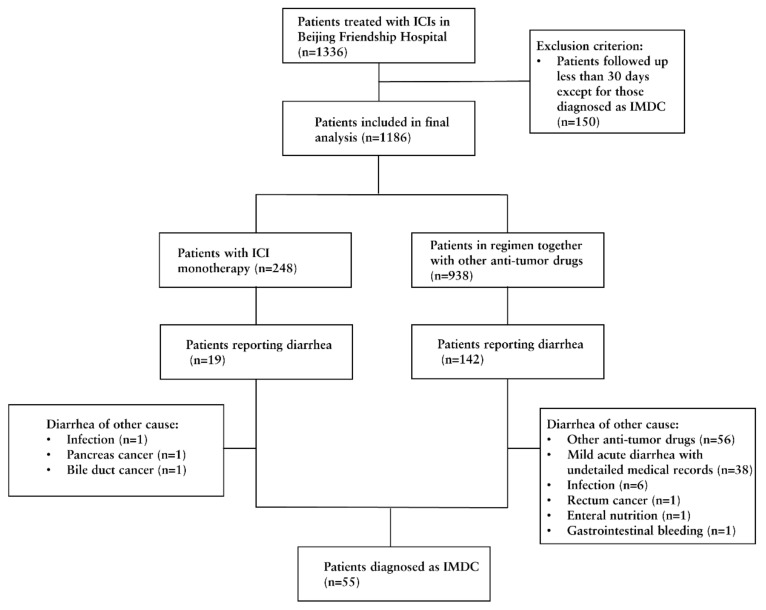
Flowchart of our study. Abbreviation: ICIs, Immune Checkpoint Inhibitors; IMDC, Immune Checkpoint inhibitor-associated Diarrhea and Colitis.

From May 2015 to August 2022, 1336 patients with solid tumors were treated with immune checkpoint inhibitors at Beijing Friendship Hospital. Among them, 1186 patients who were followed for a minimum of 30 days were included in our study (Figure 1). The median duration of follow-up time was 217 (110, 387) days. Of the 1186 patients, 1157 received PD-1 inhibitors, 29 received PD-L1 inhibitors, and none received CTLA-4 inhibitors (Table 1). The incidence of immune-mediated diarrhea and colitis was found to be 4.6% and 0.67%, respectively. The median time from initiation of immune checkpoint inhibitor therapy to the onset of diarrhea was 51 (21, 147) days. A subgroup analysis was performed among patients who received monotherapy with immune checkpoint inhibitors, revealing a median time from therapy initiation to diarrhea onset was 79 (43, 289) days.

### 3.2. Comparison of Clinical Characteristics Among IMDC, Diarrhea of Other Reasons and Non-Diarrhea Groups

As shown in Table 1, no statistically significant differences were observed in terms of gender, history of smoking, or alcohol use among the three groups. However, patients with diarrhea were generally older than individuals in the other groups (66 vs. 68 vs. 65, *p* = 0.043). Additionally, the proportion of autoimmune disease was significantly higher in patients with IMDC (3.6% vs. 3.8% vs. 1.3%, *p* = 0.047). Furthermore, patients with tumors in the digestive system were more likely to experience IMDC (72.7% vs. 54.7% vs. 49.3%, *p* < 0.001). The specific tumor classifications and corresponding numbers are displayed in Supplementary Figure S1.

Regarding laboratory examination results, patients with diarrhea had a higher level of baseline TBil (12.2 vs. 12.9 vs. 11.1, *p* = 0.019). No other pretreatment laboratory examinations included in our study yielded statistically significant differences among the groups.

There was no significant difference in the type of ICIs between patients with IMDC and those without. However, patients with diarrhea received fewer total cycles of ICI compared to those without diarrhea (2 vs. 2 vs. 3, *p* < 0.001).

In terms of concomitant medications, patients with IMDC had a lower proportion of regimens, including platinum (36.4% vs. 48.1% vs. 53.0%, *p* = 0.041) and targeted drugs (16.4% vs. 29.2% vs. 22.9%, *p* = 0.015), compared with patients without diarrhea. The use of other drugs, including 5-Fu/capecotabine, taxanes, irinotecan, PPI, antibiotics, NSAIDs, and corticoids, did not show a significant association with IMDC.

### 3.3. Independent Predictors for IMDC

Multivariable regression analysis identified digestive system tumors (odds ratio [OR] 2.79, 95% confidence interval [CI] 1.42–5.75, *p* = 0.004) as a risk factor for IMDC. Conversely, platinum-based chemotherapy (OR 0.41, 95% CI 0.21–0.78, *p* = 0.008) and targeted drugs (OR 0.25, 95% CI 0.07–0.65, *p* = 0.011) were found to be potentially protective factors against IMDC (Table 2).

### 3.4. Patients with Tumor of Digestive System Were Prone to IMDC

**Figure 2:**
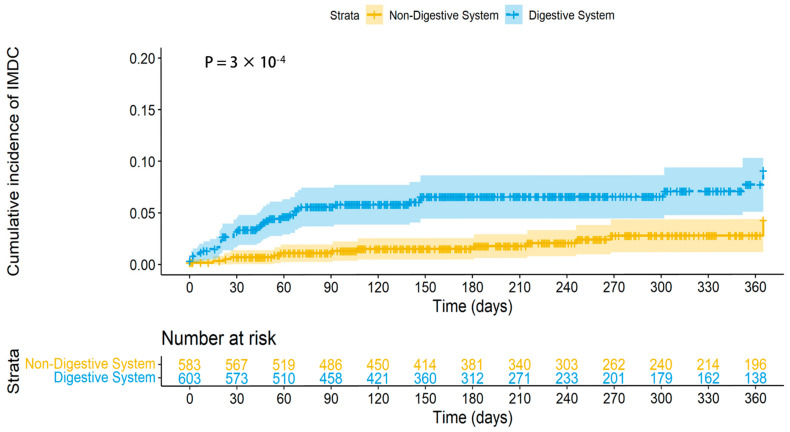
Cumulative incidence curve of IMDC stratified by tumor of the digestive system and non-digestive system. The *p*-value was calculated using a Log-rank test. Abbreviation: IMDC, Immune Checkpoint inhibitor-associated Diarrhea and Colitis.

The Log-rank test further demonstrated that patients with digestive system tumors treated with PD-1/PD-L1 inhibitors had a higher incidence of IMDC (*p* < 0.001, Figure 2).

### 3.5. Immune-Related Adverse Events (irAEs) and ICI Discontinuation

In our study cohort, out of 1186 patients, 45 (3.8%) discontinued immune checkpoint inhibitor (ICI) treatment due to irAEs. As shown in Table 3, the lung, heart, and colon were identified as the most common sites of irAEs, resulting in treatment discontinuation, accounting for 35.6%, 20.0%, and 15.6%, respectively. More specifically, among the 55 IMDC patients, 7 (12.7%) discontinued treatment.

## 4. Discussion

In this study, the incidence and risk factors associated with IMDC were examined among Chinese patients treated with PD-1 or PD-L1 inhibitors. The overall incidence rate of IMDC was 4.6%, with ICI-associated colitis occurring in 0.67% of cases. After univariable analysis and subsequent confirmation via multivariable modeling, digestive system tumors were identified as independent risk factors for IMDC. However, the data did not provide substantial evidence to support the notion that PPI, antibiotics, NSAIDs, and glucocorticoids serve as either protective or risk factors for IMDC. Furthermore, ICI-associated colitis was the third most prevalent cause leading to ICI discontinuation in our cohort, accounting for 15.6%. It is noteworthy that in our clinical practice, no patients received anti-CTLA4 antibodies. This limitation should be considered when interpreting the results, as they specifically apply to patients treated with PD-1/PD-L1 inhibitors.

Immune checkpoints serve as pivotal molecules in regulating immune responses to prevent autoimmunity, and their blockade can disrupt immune tolerance in normal tissues, resulting in immune-related adverse events [14]. In the context of ICI-associated colitis, the involvement of cytokines and gut microbiome has been emphasized in its pathogenesis [15]. According to a meta-analysis conducted by Nielsen, D. L based on clinical trials, the incidence of ICI-associated diarrhea was found to be 10.69% for PD-1 inhibitors and 7.97% for PD-L1 inhibitors, while the incidence of colitis was reported to be 1.18% and 0.25%, respectively [4]. Another meta-analysis, based on observational studies with a total population of 5727 patients, reported an incidence of IMDC of 4.1% [16]. It is important to note that these data primarily represent the Caucasian population. Furthermore, Farha, N.’s study highlighted that Caucasian individuals were more prone to developing ICI-associated colitis compared to African Americans, suggesting that race may play a role in the incidence of IMDC [5]. Consequently, previous reports may not accurately reflect the occurrence of IMDC in the Chinese population. To the best of our knowledge, our study is the first to specifically investigate the incidence rate of IMDC in the Chinese population. Our findings suggest that the incidence of IMDC in the Chinese population may be lower in comparison to the Caucasian population. A recent study from Korea reported an incidence rate of 3.5% for IMDC in the Korean population. The main types of ICIs used in that study, as well as the sample size and the definition of IMDC, are consistent with our research [17]. Combined with our findings, this further supports the notion that the incidence of IMDC may be lower in Asian populations compared to Caucasian populations.

In addition, our data support the notion that digestive system tumors serve as a risk factor for the development of IMDC. Patients with digestive system tumors accounted for 50.8% of all the cases in our study. These patients exhibited a significantly higher likelihood of developing IMDC (OR = 2.79, *p* = 0.004) in comparison to those with other tumor types after multivariable analysis. As described in the Methods section, we excluded all the patients with pre-existing diarrhea prior to initiating ICI treatment, thereby minimizing the occurrence of tumor-related diarrhea. The median duration from ICI initiation to the onset of diarrhea was found to be 51 days, which may be insufficient for significant tumor progression. Few studies directly reported the variations in the incidence of IMDC based on tumor type or location. Through a systemic review and meta-analysis of prospective monotherapy trials of ICIs, Khoja, L concluded that melanoma patients exhibit a higher incidence of IMDC compared with non-small-cell lung cancer patients [6,18], which also supports the incidence difference among tumor types. However, when comparing incidence across different cohorts, caution is warranted due to inherent differences in patient characteristics and selection criteria.

Previous studies found that antibiotics [7,8], NSAIDs [10], and PPI [9] may increase the risk of IMDC. However, our findings did not support these prior observations. Regarding antibiotics, Abu-Sbeih, H.’s study demonstrated that antibiotics use following ICI initiation was a risk factor for IMDC; however, this association was not supported by the findings of our study. The difference in ICI class may have influenced the results, as approximately half of the patients in Abu-Sbeih, H.’s study received anti-CTLA4 therapy or combination therapy [7]. Additionally, Abu-Sbeih, H.’s study seemed to report a much higher IMDC incidence than other studies [4,16], with 65% for patients without antibiotic exposure and 100% for patients receiving antibiotics after ICI initiation, without a clear definition of IMDC and adjustment of multivariable analysis. In terms of NSAIDs, a significant difference between our study (n = 1186) and the previous study (n = 73) [10]. For PPI, the definition of PPI exposure was restricted to within 3 months before ICI initiation in Yin, J.’s study [9], whereas our study only collected the information after ICI initiation, rendering the results incomparable. Despite glucocorticoids being recommended as the first-line treatment regimen for immune checkpoint inhibitor-associated colitis [11,12], their use for anti-allergy purposes prior to anti-tumor drugs in our study did not demonstrate any significant influence on the incidence of IMDC. Further research is required to investigate these potential risk factors in a more comprehensive manner, utilizing a larger sample size and clearly defining the exposure factors and outcomes.

Surprisingly, our data suggested that platinum-based chemotherapy and targeted drugs may serve as protective factors for IMDC. Currently, there are no other studies reporting a reduction in the incidence of IMDC with these two types of drugs. Additionally, a discrepancy in follow-up endpoints was noted, with patients experiencing IMDC having a shorter median follow-up time (51 days vs. 217 days). This discrepancy resulted in a greater likelihood of concurrent medication use during immune checkpoint inhibitor treatment in those without IMDC. When adjusting the follow-up endpoint to the last follow-up in our hospital for patients with IMDC, there was no significant difference in the concurrent use of platinum (60.0% vs. 53.0%, *p* = 0.227) and targeted drugs (34.5% vs. 22.9%, *p* = 0.112). However, in light of the frequent adverse reactions observed with platinum agents, such as bone marrow suppression and decreased white blood cell count [19], we hypothesize that these reactions may, to some degree, mitigate the immune activation effects brought by immune checkpoint inhibitors. Hence, further investigation is essential to confirm the validity of our findings. For targeted drugs, aside from the different follow-up times, the lack of waxing and waning patterns of increased bowel movements and/or loose stools correlating with treatment cycles makes it difficult to distinguish it from IMDC. Moreover, given the much higher reported incidence of diarrhea caused by targeted drugs compared to PD-1/PD-L1 [4,13], for patients in the regimen of both drugs, only those with endoscopic and pathologic evidence, or those recovered after immunosuppressive therapy were diagnosed as IMDC, which may underestimate the incidence of IMDC in these patients. However, targeted drugs were not commonly used in our cohort, which may only have a slight influence on the incidence of IMDC in the total study population.

According to the AGA guideline, moderate to severe IMDC necessitates anti-inflammatory treatment and discontinuation of ICIs [12]. In our cohort, the colon was the third most common site of irAE, resulting in ICI discontinuation, followed by the lung and heart. Few studies directly report the rate of ICI discontinuation because of irAEs; however, ICI delay and discontinuation was recommended as the first step of management of any grade of pneumonitis [20], possible myocarditis [21] and IMDC (grade ≥ 2) [12] according to separate clinical guidelines. Systematic reviews have reported an incidence of all grade pneumonitis of approximately 3% [22], myocarditis of 0.46% [23], and IMDC (grade ≥ 2) of 0.19–1.18% [4], which are similar to our results both in absolute relative estimate. Given its high incidence, IMDC impacts not only primary tumor therapy but also patient safety. Therefore, the recognition and intervention of IMDC risk factors are of great importance in clinical practice.

To prevent the occurrence of IMDC, researchers have increasingly focused on the microbiome’s role in regulating immune responses. The gut microbiota is thought to be essential in maintaining host immune homeostasis and may affect the efficacy and side effects of ICIs [24,25]. For instance, beneficial bacteria such as *Faecalibacterium prausnitzii* have been shown to reduce ICI-induced colitis while enhancing anti-tumor immune responses [25]. Moreover, fecal microbiota transplantation (FMT), a method for modulating the gut microbiome, has demonstrated the potential to alleviate IMDC symptoms and improve treatment outcomes in certain cases [26,27].

This study has some limitations. It is a retrospective cohort study with potential data deficiencies. In clinical practice, the recording of adverse events may not be timely or comprehensive, particularly for mild cases, despite a thorough review of patients reporting diarrhea following ICI initiation. Moreover, the collection of concomitant medications was limited to those administered after ICI initiation, thus obscuring the medication history prior to ICI use, which may also introduce confounding factors. Our study did not evaluate specific chemotherapy regimens in relation to IMDC incidence, as no fixed regimen was used due to the diverse tumor types included. Therefore, it is imperative to conduct additional prospective studies to validate our findings.

## 5. Conclusions

In conclusion, the incidence of IMDC within the Chinese population was identified at 4.6%, ranking it as the third most commonly observed immune-related adverse event leading to the discontinuation of immune checkpoint inhibitors. Remarkably, risk and protective factors for IMDC were independently constituted by tumors originating from the digestive system and the utilization of platinum-based agents. Conversely, the incidence of IMDC was not significantly impacted by using PPIs, antibiotics, NSAIDs, and glucocorticoids.

## Figures and Tables

**Table 1:** Comparison of clinical characteristics among IMDC, diarrhea of other causes, and non-diarrhea groups.

	IMDC (n = 55)	Other Causes (n = 106)	Non-Diarrhea (n = 1025)	*p*-Value ^a^	FDR
Age, median (quantile)	66 (59, 74)	68 (63, 72)	65 (59, 71)	0.043	0.109
Male, n (%)	42 (76.4%)	74 (69.8%)	755 (73.7%)	0.612	0.714
Smoking, n (%)	25 (45.5%)	33 (31.1%)	384 (37.5%)	0.184	0.343
Alcohol use, n (%)	17 (30.9%)	21 (19.8%)	266 (25.9%)	0.241	0.422
Autoimmune disease, n (%)	2 (3.6%)	4 (3.8%)	13 (1.3%)	0.047	0.110
Cancer Type					
Digestive tract	29 (52.7%)	39 (36.8%)	381 (37.2%)	<0.001	<0.001
Colorectal, n (%)	6 (10.9%)	9 (8.4%)	100 (9.6%)		
Non-Colorectal, n (%)	23 (41.8%)	30 (28.3%)	281 (27.5%)		
Hepto-bilio-pancreatic	11 (20.0%)	19 (17.9%)	124 (12.1%)	<0.001	<0.001
Pancreas, n (%)	1 (1.8%)	2 (1.9%)	18 (1.8%)		
Liver, n (%)	7 (12.7%)	10 (9.4%)	83 (8.1%)		
Gallbladder and Bile duct, n (%)	3 (5.5%)	7 (6.6%)	23 (2.2%)		
Others, n (%)	15 (27.3%)	48 (45.3%)	520 (50.7%)	<0.001	0.013
Laboratory Examinations					
TBil, median (quantile)	12.2 (8.5, 17.3)	12.9 (9.3, 16.9)	11.1 (8.2, 15.3)	0.019	0.066
Alb, median (quantile)	35.6 (30.8, 40.2)	38.4 (34.8, 40.6)	37.2 (33.9, 40.0)	0.083	0.166
SCr, median (quantile)	68.1 (52.0, 84.3)	66.3 (57.8,78.4)	65.2 (55.9, 77.4)	0.669	0.720
WBC, median (quantile)	5.3 (3.7, 6.4)	6.0 (4.7, 8.5)	5.8 (4.4, 7.6)	0.055	0.118
HGB, median (quantile)	125.0 (106.5, 136.0)	120.0 (111.0, 133.0)	119.5 (108.0, 133.2)	0.662	0.720
PLT, median (quantile)	195.0 (147.5, 260.0)	194.0 (145.8, 235.8)	200.0 (149.0, 256.0)	0.391	0.539
CRP, median (quantile)	15.9 (3.6, 37.7)	8.8 (2.8, 41.0)	10.8 (2.5, 38.1)	0.862	0.862
LDH, median (quantile)	187.0 (142.0, 260.0)	169.5 (149.5, 219.2)	171 (142, 224.8)	0.322	0.501
ICI type				0.342	0.504
PD-1, n (%)	53 (96.4%)	102 (96.2%)	1002 (97.8%)		
PD-L1, n (%)	2 (3.6%)	4 (3.8%)	23 (2.2%)		
Total ICI cycles, median (quantile) Concomitant medications	2 (2, 4)	2 (1, 4)	3 (2, 6)	<0.001	0.002
Other anti-tumor drugs, n (%)	39 (70.9%)	95 (89.6%)	793 (78.1%)	0.006	0.028
5-Fu/capecotabine	17 (30.9%)	22 (20.7%)	226 (28.3%)	0.282	0.464
Irinotecan	3 (4.0%)	5 (4.7%)	12 (1.2%)	0.024	0.075
Taxanes	5 (9.1%)	38 (35.8%)	106 (10.3%)	<0.001	<0.001
Platinum	20 (36.4%)	51 (48.1%)	543 (53.0%)	0.041	0.109
Targeted drugs	9 (16.4%)	31 (29.2%)	235 (22.9%)	0.015	0.060
PPI, n (%)	31 (56.4%)	67 (63.2%)	620 (60.5%)	0.698	0.724
Antibiotics, n (%)	20 (36.4%)	47 (44.3%)	418 (40.8%)	0.609	0.714
NSAIDs, n (%)	13 (23.6%)	34 (32.1%)	302 (29.5%)	0.536	0.682
Glucocorticoid, n (%)	38 (69.1%)	77 (72.6%)	779 (76.0%)	0.404	0.539

Abbreviations: IMDC, Immune Checkpoint inhibitor-associated Diarrhea and Colitis; n, number. TBil, Total Bilirubin; Alb, Albumin; SCr, Serum Creatinine; WBC, White Blood Cell; HGB, Hemoglobin; PLT, Platelet; CRP, C-Reaction Protein; LDH, Lactate Dehydrogenase; ICI, Immune Checkpoint Inhibitor; PD-1, Programmed cell Death protein 1; PD-L1, Programmed cell Death protein Ligand 1; FDR, False Discovery Rate. ^a^ *p*-values were calculated by Mann–Whitney U test, χ^2^ test or Fisher’s exact test.

**Table 2:** Multivariable logistic regression model of potential risk factors of IMDC.

Exposure Factors	OR	Confidant Interval	*p*-Value
Age	0.99	(0.97, 1.03)	0.739
Male	1.70	(0.80, 4.06)	0.196
Autoimmune disease	3.78	(0.52, 16.9)	0.117
Tumors of the digestive system	2.79	(1.42, 5.75)	0.004
TBil	1.00	(0.97, 1.01)	0.744
ICI cycles	1.01	(0.94, 1.07)	0.747
Irinotecan	1.80	(0.09, 11.22)	0.596
Taxanes	0.84	(0.28, 2.04)	0.726
Platinum	0.41	(0.21, 0.78)	0.008
Targeted drugs	0.25	(0.07, 0.65)	0.011

Abbreviations: IMDC, Immune Checkpoint inhibitor-associated Diarrhea and Colitis; OR. Odds Ratio; TBil, Total Bilirubin; ICI, Immune Checkpoint Inhibitor.

**Table 3:** The proportion of IMDC among irAEs resulting in ICI discontinuation.

Sites of irAEs Resulting in ICI Discontinuation	Number of Cases	Proportion
Lung	16	35.6%
Heart	9	20.0%
Colon	7	15.6%
Liver	4	8.9%
Endocrine system	3	6.7%
Skeletal muscle	3	6.7%
Derma	2	4.4%
Joint	1	2.2%

Abbreviations: IMDC, Immune Checkpoint inhibitor-associated Diarrhea and Colitis; irAEs, immune-related Adverse Events.

## Data Availability

The datasets analyzed during the current study are available from the corresponding author upon reasonable request.

## Supplementary Materials

The following supporting information can be downloaded at: https://www.mdpi.com/article/10.3390/medicina61020353/s1, Figure S1: Classification of tumor locations and corresponding patient numbers among enrolled patients.

## Abbreviations

ICIs: immune checkpoint inhibitors; IMDC, Immune Checkpoint inhibitor-associated Diarrhea and Colitis; PPIs, Proton Pump Inhibitors; NSAIDs, Nonsteroidal Anti-Inflammatory Drugs; irAEs, immune-related Adverse Events; CTLA4, Cytotoxic T-Lymphocyte Antigen 4; PD-1, Programmed Death Protein 1; PD-L1, Programmed Death Ligand 1; NSCLC, Non-Small-Cell Lung Cancer; EMR, Electronic Medical record; TBil, Total Bilirubin; Alb, Albumin; SCr, Serum Creatinine; WBC, White Blood Cell; HGB, Hemoglobin; PLT, Platelet; CRP C-Reaction Protein; LDH, Lactate Dehydrogenase.
