# Supplementary material for: Incidence of and Risk Factors for Anti-PD-1/PD-L1- Associated Diarrhea and Colitis: A Retrospective Cohort Study of the Chinese Population
Source: Medicina (Kaunas). 2025 Feb 18;61(2):353. doi: 10.3390/medicina61020353 (PMC11857649; doi:10.3390/medicina61020353)
Supplement: Supplementary file 1 [file medicina-61-00353-s001.zip › medicina-3435282-supplementary.pdf]

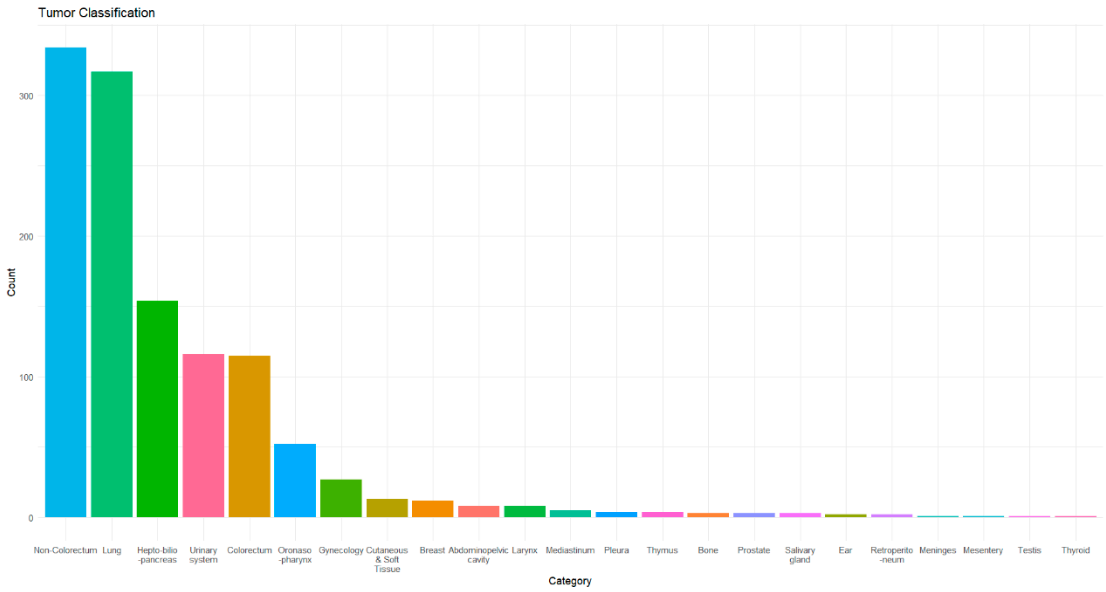

Supplementary Figure S1. Classification of tumor locations and corresponding patient numbers among enrolled patients.
